# Pan-Nematoda Transcriptomic Elucidation of Essential Intestinal Functions and Therapeutic Targets With Broad Potential

**DOI:** 10.1016/j.ebiom.2015.07.030

**Published:** 2015-07-29

**Authors:** Qi Wang, Bruce A. Rosa, Douglas P. Jasmer, Makedonka Mitreva

**Affiliations:** a McDonnell Genome Institute, Washington University School of Medicine, St. Louis, MO, USA; b Department of Veterinary Microbiology and Pathology, Washington State University, Pullman, WA, USA; c Division of Infectious Disease, Department of Internal Medicine, Washington University School of Medicine, St. Louis, MO, USA

**Keywords:** Nematode intestine, Molecular functions, Intestinal protein families, Therapeutic targets

## Abstract

The nematode intestine is continuous with the outside environment, making it easily accessible to anthelmintics for parasite control, but the development of new therapeutics is impeded by limited knowledge of nematode intestinal cell biology. We established the most comprehensive nematode intestinal functional database to date by generating transcriptional data from the dissected intestines of three parasitic nematodes spanning the phylum, and integrating the results with the whole proteomes of 10 nematodes (including 9 pathogens of humans or animals) and 3 host species and 2 outgroup species. We resolved 10,772 predicted nematode intestinal protein families (IntFams), and studied their presence and absence within the different lineages (births and deaths) among nematodes. Conserved intestinal cell functions representing ancestral functions of evolutionary importance were delineated, and molecular features useful for selective therapeutic targeting were identified. Molecular patterns conserved among IntFam proteins demonstrated large potential as therapeutic targets to inhibit intestinal cell functions with broad applications towards treatment and control of parasitic nematodes.

## Highlights

• The intestine is an ideal target for therapeutics to control parasitic nematodes.
• This is a pioneering pan-phylum study of the intestinal transcriptome/proteome in the Nematoda.
• Conserved intestinal cell functions were delineated for the phylum clades and species of nematodes.
• The possibility for selective targeting for therapeutic purposes is discussed.

Using multi-omic data integration and evolutionary considerations, we derived a natural history of births, deaths and variation in family members for over 10,000 intestinal protein families conserved among ten nematode species. The development of therapeutics is explored in relation to conserved intestinal cell functions and molecular features specific to the parasite proteins.

## 1 Introduction

Parasitic nematodes comprise a major group of pathogens that infect nearly one third of the human population, and compromise, or threaten, the health and productivity of most agricultural animals and plants throughout the world. The selection of anthelmintics (nematicides) that can be used for treatment and control of these pathogens is relatively limited, and acquired resistance to anthelmintics by parasitic nematodes is a growing problem (Albonico et al., 2004; Awadzi et al., 2004; Bourguinat et al., 2008; Osei-Atweneboana et al., 2007; Vercruysse et al., 2012). Consequently, a need exists to identify new parasite targets for therapeutic intervention and the nematode intestine provides a tissue to investigate for this purpose. The intestine of parasitic nematodes, and nematodes in general, is sited at an internal interface that is continuous with the outside environment. As such, the intestine is accessible to anthelmintics that can interfere with intestinal cell functions essential for survival of nematodes. The intestine has a relatively simple tubular design formed by a single cell layer of intestinal cells that resemble polarized epithelial cells (Yin et al., 2008). This single cell layer serves as a physical separation between the environment and the pseudocoelomic body cavity. Evidence indicates that a wide range of functions involved in nutrient acquisition (McGhee, 2007; Yin et al., 2008; Jasmer et al., 2015; Rosa et al., 2015), physiological homeostasis and basic defense against environmental toxins (Park et al., 2001) are all sited at the apical intestinal membrane. Intestinal cells are also a primary site for synthesis of yolk proteins that are eventually incorporated into ova (Chotard et al., 2010). Nematode intestinal cells Protein families (orthologous groups) were defined utilizing the Markov cluster algorithm (Enright et al., 2002) using the OrthoMCL also have clear importance as targets for anthelmintic therapies related to vaccines (Jasmer et al., 1993; Smith, 1993; Pearson et al., 2010), contemporary anthelmintics (Jasmer et al., 2000) and biotoxins (Wei et al., 2003). Nevertheless, the limited knowledge on biological properties of nematode intestinal cells and the extent to which those properties are conserved among parasitic species has impeded research aimed at developing new therapeutic methods directed at this tissue. Indeed, the challenges related to research on the nematode intestine extend to virtually every tissue in parasitic nematodes. Hence, methods related to solving challenges related to the intestine have application to other tissues of nematode pathogens.

Nematode species display remarkable versatility in relation to the diverse trophic niches that they occupy and display substantial diversity at morphological levels of the intestine (Munn and Greenwood, 1984). Hence, while many basic intestinal cell characteristics are likely to be broadly conserved, other characteristics may markedly vary among different nematode lineages and species. Orthologous protein families conserved among phylogenetically diverse nematode species have been investigated (Mitreva et al., 2011), while only modest attempts have been made to deduce characteristics of nematode intestinal cells that are conserved among, and potentially ancestral to, all nematodes (Yin et al., 2008).

We initially sought to develop computational platforms to distinguish between subsets of intestinal proteins that are essential to all nematodes and those that may be related to success of individual lineages or species. A comprehensive assessment of this kind will provide a knowledge base that will drive development of research hypotheses and support research to elucidate biological functions essential for the survival of many, or all parasitic nematodes.

**Fig. 1:**
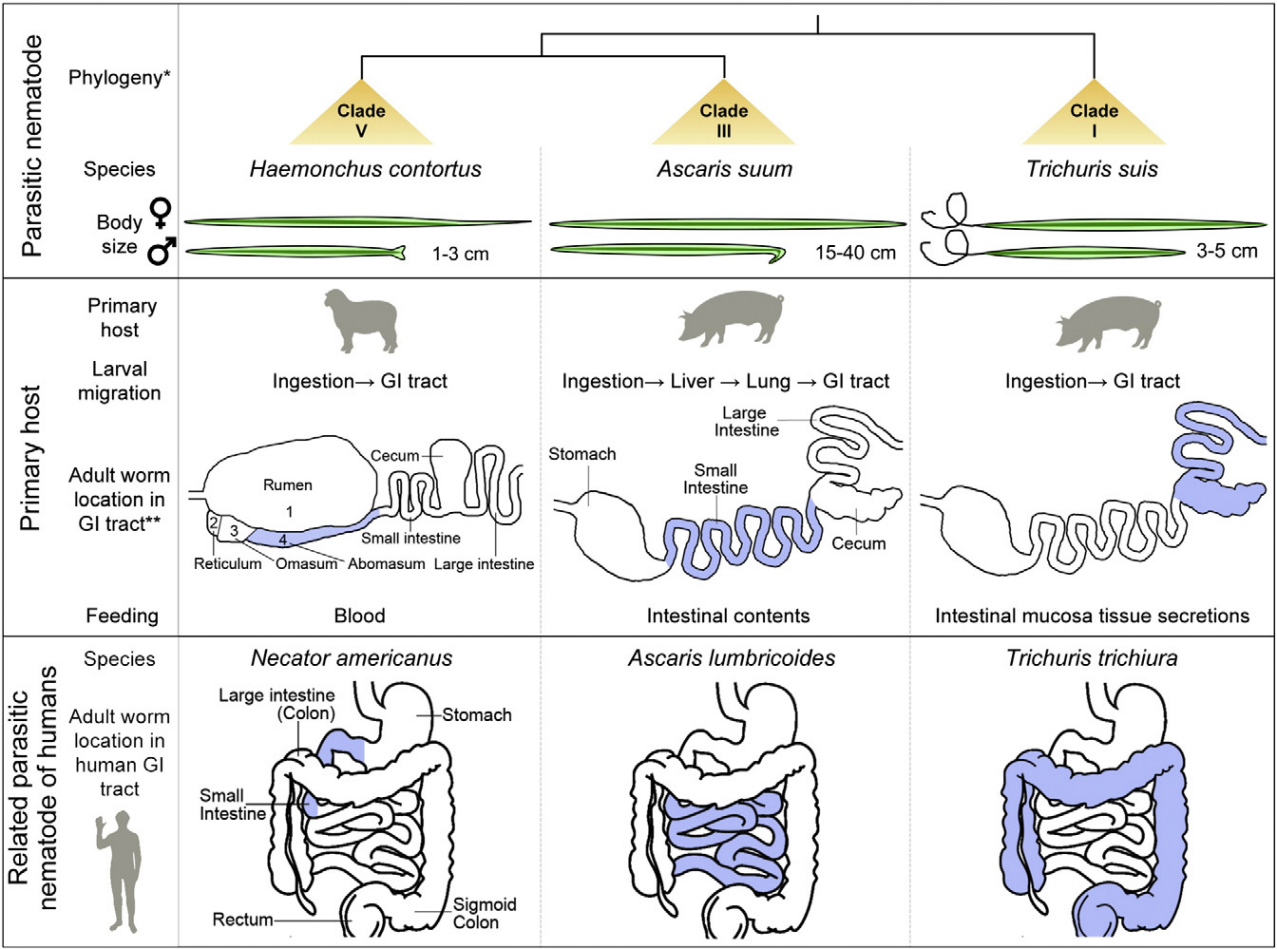
Characteristics of core species: phylogeny, host location and trophic ecology. *Phylogeny based on Blaxter et al., 1998. ** GI tract, gastro-intestinal tract.

The selection of species investigated was critical for success in this research (Fig. 1). Using RNA from dissected intestines we directly identified the genes expressed in the adult intestine of three parasitic nematode species of livestock: *Trichuris suis*, *Ascaris suum* and *Haemonchus contortus*. Each of these is a soil transmitted pathogen with model applications to other phylogenetically related pathogens of humans and animals. Each of these species, referred to here as ‘core species’, is a member of a different and diverse nematode taxonomic clade (I, III and V, respectively (Blaxter et al., 1998)), and each core species occupies a distinct trophic niche (epithelial layer of the swine cecum, swine small intestinal lumen and blood feeder at the abomasal mucosa of small ruminants, respectively), likely requiring diverse adaptations for success (Fig. 1). Therefore, intestinal cell proteins and functions conserved among these phylogenetically and biologically diverse nematodes are likely to reflect basic features ancestral to many or all nematodes. Beyond these considerations, the relatively large size of the adult worms of each of the core species supports dissection to obtain intestinal tissue and RNA, which is otherwise difficult for most nematode species.

We sought to identify nematode intestinal cell characteristics based on direct genome-wide intestinal transcript evidence for species that span the phylum Nematoda. Transcript expression data was coupled with predicted or known intestinal proteins to deduce functional characteristics that are broadly conserved among (and potentially ancestral to) all nematodes. A platform was also established to assess variation in intestinal cell characteristics that may have contributed to the diversity among nematode lineages and species, at a whole-protein sequence level. Comparisons of deduced functions assessed the determinants of evolutionary success at the phylum, lineage and species levels. Data distinguishing conserved and variable characteristics were then used to explore molecular patterns of proteins that may have broad application to chemical intervention of nematode intestinal cell functions. Importantly, the research also established a general approach that can be used to derive proteins and functions of ancestral importance to other nematode tissues such as neuromuscular, hypodermal and reproductive tissues, to name a few.

## 2 Materials & Methods

### 2.1 Ethics Statement

The research involving use of swine was reviewed and approved by the Washington State University Institutional Animal Care and Use Committee, protocol #04097-004, approved on 12/19/2013. Guidelines are provided by the Federal Animal Welfare Act, USA.

### 2.2 Data Collection

Whole deduced proteome data (proteomes) from 15 eukaryotic species (10 nematodes and 3 hosts and 2 outgroups) were downloaded from various sources (Supplementary materials and methods). The final dataset contained of 248,475 sequences (Table S1).

### 2.3 Parasite Material and RNA Extraction and RNA-seq Data Generation

Adult *A. suum* and *T. suis* were obtained from swine infected as weanling pigs, 60–70 or 50 days post-infection with eggs (respectively), and adult *H. contortus* were obtained at 28 days post-infection from infected lambs. Intestines were dissected from freshly isolated worms, and samples from male and female worms were prepared separately for each species. Non-normalized cDNA was used to construct multiplexed illumina paired-end small fragment libraries. Multiple indexed libraries were pooled together and loaded into one lane of a HiSeq2000 version 3 flow cells. 2 × 101 bp read pairs were generated for each sample. Details on preparation procedures, RNA-seq cleaning, mapping and expression level calculation is described in detail in the Supplementary materials and methods.

### 2.4 Protein Family Definition and Identification of Intestinal Protein Families

**Fig. 2:**
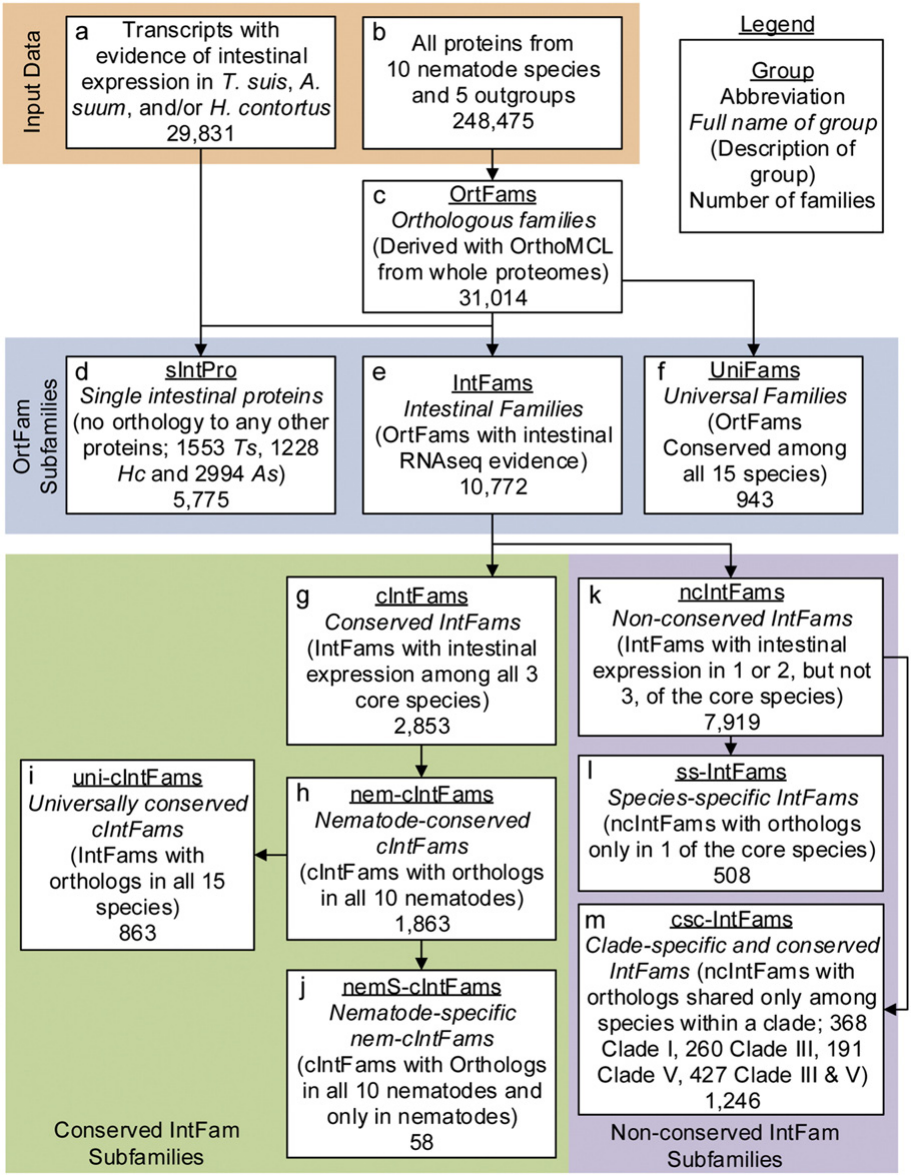
Derivation of the intestinal protein families (IntFams) in this study.

Protein families (orthologous groups) were defined using the OrthoMCL package (Fischer et al., 2011) with an inflation factor 1.5, based on the organisms' complete deduced proteomes (Table S1). Each protein family consists of at least two proteins from one or more species (Table S2). Protein families having members in all 15 species were defined as universal families (UniFam). The union of all the proteins expressed in the intestines from the RNA-seq data of any of the three species studied was identified in the protein families, and these protein families they belong to were defined as intestinal protein families (IntFam) and their members were the subject of this study (Fig. 2). Protein family death and birth events, as well as duplication and deletion events of protein sequences within protein families, were identified as previously described (Wang et al., 2012) (Supplementary materials and methods).

### 2.5 Functional Annotation and Enrichment

Interproscan (Quevillon et al., 2005) was used to determine associations of proteins to Gene Ontology (GO) terms. Interproscan also identified predicted Interpro (IPR) domains found in each gene. GO term/IPR domain enrichment for the each of the terms identified within the protein families of interest was determined using a non-parametric binomial distribution test with a 0.05 p-value cutoff for significance, after false discovery rate (FDR) population correction. The association of a GO term/IPR domain in a subset of protein families was determined as follows (Fig. S1): i) Determine the IPR/GO association for each protein; ii) for the subset of protein families of interest, identify the protein members and species of origin; iii) for each protein family within a species, if 50% or more of its proteins for a species has a IPR/GO term association, the function was assigned to all proteins within that species. Then, if the family has 50% or more species with a certain IPR/GO, then the protein family is associated with this term; and iv) the association for the IPR/GO term in the subset of protein families will be the total number of protein families associated with that term. Based on our evaluations at intervals of 10%, the 50% threshold (percent of protein family members being annotated with one term) produced optimal differences among different clades and lineages of the phylogenetic tree (Fig. S2). For each of the three core species, some of the proteins in the transcriptomes were not included within the protein families built from proteomes (i.e. orphan proteins; “singletons”). Each of these singletons was considered to be a unique new family (i.e. family with just one member). GO term/IPR domain enrichment was also assessed in these species-specific protein families, with all of the other protein families with more than one member included as background. For each of the three core species, some of the proteins in the transcriptomes were not included within the protein families built from proteomes (i.e. orphan proteins; “singletons”). Each of these singletons was considered to be a unique new family (i.e. family with just one member). GO term/IPR domain enrichment was also assessed in these species-specific protein families, with all of the other protein families with more than one member included as background.

### 2.6 Indel Detection and Structure Localization

Nematode-specific insertions and deletions in the protein sequences were identified as previously reported (Wang et al., 2009), as described in detail in the Supplementary materials and methods. Nematode proteins were compared (BLAST with threshold 1e − 05, 35% identity at over 50% fraction of length) to 257,330 sequences (including multiple chains for a single protein) with known tertiary structure downloaded from the Protein Data Bank (PDB) (Bernstein et al., 1977) to identify homologs. SiteHound (Ghersi and Sanchez, 2009) was used to identify any potential ligand binding sites based on the top PDB hit structure. The alignment of the NemFam vs. RefFam sequences was subsequently mapped to the PDB structure sequence. If a sizable indel (> 4 amino acid (AA)) was detected within a short proximity (within 3 AA range in sequence) to the top three ligand binding sites (by comparing their composite residues to the indel location), the indel was classified as a target site of interest. Modeling of protein structures was carried out using the I-TASSER Suite 2.1 (Roy et al., 2010). The alignments of nematode vs. reference proteins were applied as restraints of the target proteins related to the templates, with other parameters set to default.

## 3 Results

### 3.1 Direct Transcriptional Evidence Among Core Species Defines Ancestral Nematode Intestinal Proteins

To relate the intestinal cell proteins expressed from the core species (*T. suis*, *A. suum* and *H. contortus*) to proteins from other nematode species, we established a database of deduced proteomes of 15 eukaryotic species (ten nematode species, three hosts and two outgroups). The 248,475 proteins across all of the species formed 31,014 orthologous protein families (OrtFams; Fig. 2; Table S1) that were phylogenetically categorized based on species representation (Fig. 2, Group c; Table S2). The most conserved category included 943 universal protein families (UniFams), with members from all nematode, host and outgroup species (Fig. 2, Group f).

RNAseq-based transcriptional profiling defined intestinal transcriptomes and proteomes for each of the three core nematode species (> 100 million paired-end reads mapped per species), which were used to identify OrtFams containing intestinal protein families from at least one of the core species (IntFams; Fig. 2, Group e; Table S3). Intestinal transcript expression was detected for a total of 7898 *T. suis*, 11,109 *A. suum*, and 10,824 *H. contortus* genes (Table 1). The predicted intestinal proteins were associated with 5680, 7089, or 6386 IntFams within each of the three core species, respectively. In combination, the transcriptome data provided evidence for existence of 10,772 unique IntFams, of which 2853 IntFams (~ 26% (2853/10,772) off all IntFams) had transcript evidence obtained from all three of the core species (cIntFams; Fig. 2, Group g; Fig. S3). The cIntFams contained intestinal proteins that are broadly shared among species from across the Nematoda. Detection of IntFams shared between any two of the three core species (Fig. S3A) but not the third could reflect a true difference or a false negative of transcript detection for the third species (possible due to insufficient coverage). By probing the complete proteome of the third core species using IntFams with transcript support from the other two species, we identified additional potential cIntFams (Fig. S3B). However, to reduce assumptions, only the highly confident cIntFams (Fig. S3A) are considered in subsequent analyses.

The cIntFams (Group g) were further categorized into subgroups reflecting different degrees of conservation among the 15 species of nematodes, hosts and outgroups: 1863 cIntFams have orthologs in all 10 nematode species investigated, irrespective of orthologs in hosts or outgroups (nem-cIntFams; Group h), of which 58 have orthologs in all 10 nematode species but no hosts or outgroups (nemS-cIntFams; Group j). A total of 7919 IntFams lack transcript support across all three core species (ncIntFams; Group k). Among the ncIntFams, 508 are species-specific (ss-IntFams; Group i) and 1246 are clade-specific (csc-IntFams; Group m). sIntPro (Group d) are single predicted intestinal proteins (i.e., no orthologs identified) each derived from transcriptomes of only one core species, but not ascribed to an IntFam (5775). The number of proteins in total for ss-IntFams and sIntPro for any core species represents the number of proteins specific to that core species in this investigation.

Here, numerous categories of intestinal proteins were resolved relative to direct intestinal transcript evidence and conservation among nematode, host and outgroup species. Table S3 provides an easy way to navigate the taxonomic structure of each IntFam and associated functional and structural annotations.

### 3.2 cIntFams are Useful Predictors of IntFam Conservation Among Nematode Species

We hypothesized that cIntFams represent a valuable predictive tool for the identification of intestinal genes and proteins that are broadly conserved among nematodes (including small parasitic species for which the intestine cannot be dissected). To test this hypothesis, we compared the mean of the total number of nematode species (out of 10) that were represented in each cIntFam and ncIntFam. There were significantly more (p < 0.001, T-test) nematode species represented in each cIntFam (9.48 ± 0.76, n = 2853) than in each ncIntFam (4.75 ± 7.39, n = 7919) (Fig. S3C). Additionally, 65% (1863/2853) of cIntFams were detected in the deduced proteomes of all 10 nematodes species, in contrast to just 6% (454/7919) of ncIntFams. Of the 943 UniFams defined earlier (conserved in all 15 species), 863 were also cIntFams (uni-cIntFams; Fig. 2, Group i). These observations support our hypothesis that cIntFams represent broadly-conserved nematode characteristics, while also inferring that about two thirds of the cIntFams (1990) conserved within nematodes lack conservation across all of the host and outgroup species.

Furthermore, we explored the possibility of using cIntFams to delineate the intestinal proteome(s) of non-core nematode species with available genome data. Towards this goal, we predicted elements of the intestinal proteome of *Trichuris muris* (for which intestinal transcriptome information is lacking) using *T. suis* (a closely-related sister species which is also a core species in this study). The predicted *T. muris* intestinal proteome includes *T. muris* IntFam members classified into the following categories: cIntFams (2853), csc-IntFam members conserved among all clade I nematodes investigated (368), IntFams shared only between *T. suis* and *T. muris* (596) and likely other proteins not accounted for in these major groups. Lower-confidence members of this intestinal proteome include *T. muris* ncIntFams detected only in *A. suum*, *H. contortus*, or both (443). *T. muris* ssIntFams and sIntPros cannot be derived from this approach, but based on *T. suis*, could number over 1700. While not without caveats, these results illustrate how intestinal transcriptome data sets may be applied to derive prospective intestinal proteomes from a large number of parasitic nematodes of global importance to the health of humans, animals and plants.

Collectively, the results provide strong support that cIntFams represent intestinal characteristics that are broadly conserved among many, or all, nematode species (pan-Nematoda), and that they can be used to directly infer with relatively high confidence intestinal proteome sets of species too small to support intestinal dissection. Functional analysis of these broadly conserved characteristics is described below.

### 3.3 Natural History of IntFams Relative to Evolution of Species Within the Nematoda

cIntFams represent a group of IntFams with broad conservation among nematodes. Nevertheless, the majority of IntFams (7919) belonged to ncIntFams (Fig. 2, Group k), and the number of protein members within IntFams originating from different species varied. This phylogenetic diversity in IntFam members is likely to represent sources of substantial functional diversity among the Nematoda. Table S4 provides a natural history in conservation and variation for each defined IntFam relative to the collection of nematode and out-group species investigated. We conducted analysis of this variation to determine how the natural history data collectively sorts among nematodes, from a phylogenetic perspective.

#### 3.3.1 IntFam Births and Deaths

**Fig. 3:**
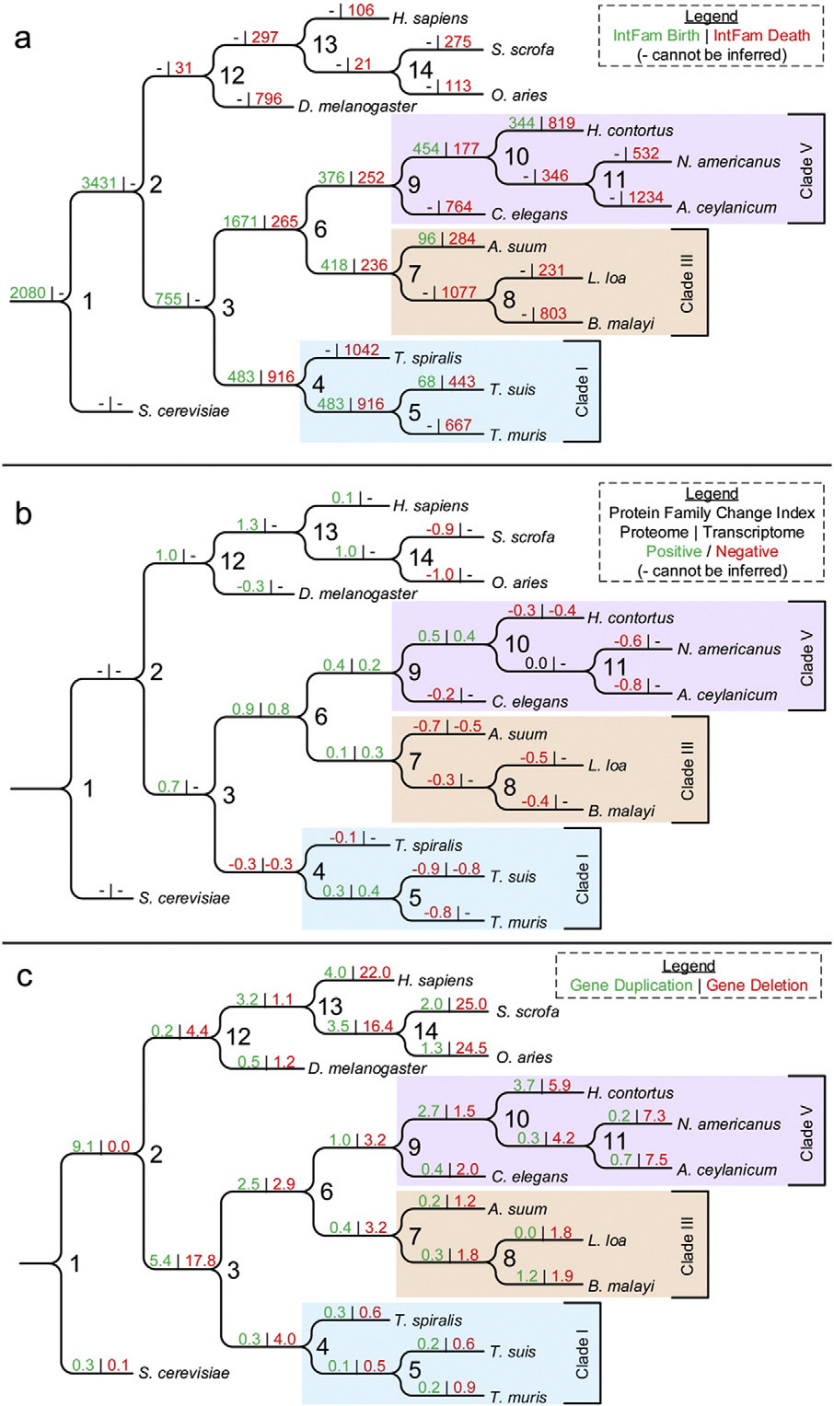
Protein family birth/death and gene duplication/loss events observed in this study. (a) Protein family birth and death events within the intestinal protein families. (b) Protein family change index (PFCI) within the protein families (proteome) and intestinal protein families (transcriptome). (c) Gene duplication and loss events for the universal families. The number or species name at each lineage corresponds to the label used in Table S5.

The first analysis followed IntFam character states (presence, birth; or absence, death) against a phylogenetic background (Fig. 3a; Table S4). Since each of the IntFams must have at least one protein member from one of the three core species to assess birth, births could not be calculated for nodes with lineages that lack a core species. In contrast, deaths reflect absence of IntFams from members of a given phylogenetic designation, which allowed calculation of deaths for all species and lineages investigated.

The most important observation from this analysis is that differences in IntFam births were observed among nodes leading to each core species, reflecting differential acquisition of IntFams and associated functions throughout the phylogenetic history of the Nematoda. While not all species are required to have the acquired IntFam (birth) in this analysis, lineage-specific IntFams that are conserved among all species in the lineage will be contained in these groups. For instance, while 755 IntFam births (Fig. 3a, node 3) were ascribed to the last common ancestor (LCA) of the Nematoda, only 58 of these IntFams were detected in all species included in this analysis (Fig. 2, Group j). Likewise, IntFam births outnumbered IntFams conserved among all species in the ascending lineage for the LCAs of clades III & V (1671; 265), clade III (418; 236) and clade V (376; 252), with the only exception of the LCA of clade I, where deaths outnumbered births (483; 916). In contrast, and expectedly, the number of IntFam births at terminal branches for each of the core species equals the species-specific IntFams. One importance here is that functions acquired at major nodes may reflect key factors in the evolutionary success of species within a given lineage, which will be discussed below.

Although IntFam births could not be calculated for branches of the tree lacking core species, evaluation of the protein family change index (PFCI) suggests that substantial IntFam births will be resolved with each additional species for which direct transcriptome data is obtained. The PFCI (defined as the logarithm of the ratio of birth to death events (Wang et al., 2012)) was evaluated for IntFams versus whole proteomes in a phylogenetic background. A high correlation (r^2^ = 0.942, p = 1.5 × 10^− 4^) was observed between the PFCI for IntFams at nodes within the Nematoda (where births and deaths could be calculated) and the PFCI for whole proteomes (Figs. 3b; S4). The results indicate that IntFams are an important source of overall diversity among nematode species and substantial additional diversity will be uncovered with addition of intestinal transcriptomes of other species.

#### 3.3.2 Variation in IntFam Members Across Phylogenetic Lineages

In addition to births and deaths of IntFams, variation in IntFam members has potential to convey functional diversity among nematode lineages and species. To gauge the pan-Nematoda impact of this variation, we confined the analysis to the 863 uni-cIntFams (Fig. 2, Group i), which had intestinal transcript evidence in all three core species, and protein members in all nematode, host and outgroup species. Proteins in this group are likely to perform basic functions common to most eukaryotic cells, and so variation in the number of members in this group provides one of the most conservative assessments of diversity that can be related to nematode IntFam members.

Only 147 of the 863 uni-cIntFams were restricted to a single protein member in each of the 15 species (single copy families), leaving 716 of the 863 uni-cIntFams with more than one protein member from at least one species (variable uni-cIntFams). The number of protein members for the variable uni-cIntFams had a maximum range from one to 23 among all 15 species and from one to 17 among nematode species. Numerous gain and loss events were observed for uni-cIntFams at all phylogenetic levels (Fig. 3c, Table S5). A total of 1838 gains and 5543 losses in nematode members were inferred for the 863 uni-cIntFams (Table S5), with a mean of 2.1 duplication and 6.4 deletion events per uni-cIntFam across these species. Hence, variation in uni-cIntFam members is a likely source of marked diversity in intestinal functions among nematode species. We can expect even more diversity related to other IntFam groups (cIntFam and ncIntFam).

Analysis of the variation in IntFam members also identified other characteristics that can distinguish nematode species. For instance, *H. contortus* had the highest total number and mean number of IntFam members among the 10 nematode species (p < 0.001; Analysis of variance, followed by Tukey's multiple comparison of means). *H. contortus* is recognized for its high genetic diversity, predominately recognized at the nucleotide level, both within and among populations (Otsen et al., 2001; Troell et al., 2006). The relatively high copy number of IntFams in *H. contortus* species identifies genetic elements on which acquisition of nucleotide polymorphisms may be facilitated.

The observed variation in IntFam members is, in part, responsible for the diversity among species of the Nematoda. Hence, relative expansion in the number of proteins within a single IntFam may reflect important adaptations or speciation events of one species compared to others. The comparatively high expansion of intestinal cathepsin B-like cysteine peptidases from *H. contortus* (Jasmer et al., 2004) provides one example. Our database provides a unique capability to identify IntFam expansion of this kind. Of the 2853 cIntFams, we identified 12 which had 10-fold or higher expansion separating the lowest to the highest counts (excluding 0) (Fig. 2, Group g; Table S3; Fig. S5). An extreme example is OrtFam_1000, containing 473 *Brugia malayi* members and 1 to 74 members among the other nematode species. The functional annotation of this IntFam was somewhat variable but consistently indicated involvement of interactions with DNA or RNA. Another example, OrtFam_1015, contained from 0 to 70 members per species, with the highest in *H. contortus*. *H. contortus* genes in this family encode Ribonuclease H-like proteins, which function in many biological processes (including DNA replication, repair and transposition and RNA interference) and have been previously shown to exhibit extensive sequence divergence across species (Majorek et al., 2014). cIntFams OrtFam_1032 and OrtFam_51034 also each exhibited more than ten-fold differences in gene numbers among nematode species and encode predicted ABC transporters, which can protect nematodes from environmental toxins and function in acquired resistance to anthelmintics in parasitic nematodes (Ménez et al., 2012; De Graef et al., 2013). As a final example, cIntFams OrtFam_1017 and OrtFam_1043 also exhibit significant interspecies variation in gene number, and encode predicted UDP-glucuronosy/UDP-glucosyltransferase and Cytochrome P450, respectively. These functions along with ABC transporters have been implicated in a three phase process of protecting nematode intestinal cells against environmental toxins (Cotton et al., 2014), and direct evidence for existence for six of these proteins has been established in the intestine of *A. suum,* of which four were inferred to be positioned on the integral intestinal membrane (Rosa et al., 2015). These examples indicate that variations in IntFam members are likely to have important physiological implications for nematode intestinal cells.

These analyses of IntFam births and deaths and gain and loss of IntFam members across species provide a composite view of the natural history of each of the IntFams directly derived from all of the core species and in relation to IntFam members discernible in predicted proteomes from each of the 15 species analyzed. The analyses quantify changes in IntFams over the course of evolution of the Nematoda, resolving proteins that likely contributed to diversification of lineages and species of nematodes, which significantly extends and complements a recent report on diversity among different compartments of the nematode intestine (Rosa et al., 2015).

### 3.4 Intestinal Cell Functions Have Central Importance to the Phylum, Lineages and Species of Nematoda

#### 3.4.1 Intestinal Cell Functions of Phylum and Lineage Importance

Intestinal functions that are broadly conserved among all nematode species investigated, and then various lineages, represent potential determinants of central importance to the evolutionary success of the Nematoda and lineages within the phylum, respectively. The predicted functions of the major categories of IntFams we have derived (cIntFams, nem-cIntFams, nemS-cIntFams, and csc-IntFams for the clades I, III, V and III & V) have significance in this context. The number of IntFams in each group ranges from 58 (nemS-IntFams) to 2853 (cIntFams). The relative importance of any individual IntFam in these groups is difficult to discern, but functional enrichment provided one approach to identify functions of potential significance (annotated functions for each IntFam of each group are shown in Table S3, and enriched functions for each group are listed in Table S6).

The nemS-cIntFams represent an especially interesting group, because functions of IntFams in this group are candidates as key determinants in the success of the Nematoda. IPR domains enriched among nemS-cIntFams provided one approach to identify significant functions. For example, five enriched IPR domains were related to Immunoglobulins, which are structural domains associated with many proteins from viruses to metazoans. A subset of OrtFams annotated with immunoglobulin domains were also enriched for terms related to fibronectin (Table S6). Fibronectins are membrane proteins that bind elements of the extracellular matrix, which may be of importance on the apical and/or basal membranes of the nematode intestine. A second example involves three “NADH:ubiquinone oxidoreductase” subunit terms. OrtFams containing these domains include OrtFam_7031, containing *Caenorhabditis elegans* W01A8.4, which has been previously described as being nematode-conserved, with a severe RNAi phenotype (Mitreva et al., 2011). A third example involved three “copper type II, ascorbate-dependent monooxygenase” terms. OrtFams containing these domains include OrtFam_6206, containing *C. elegans* T19B4.1, which is involved in sensitivity to the nematicide Aldicarb (Sieburth et al., 2005), and all OrtFams annotated with another enriched term, PHM/PNGase F-fold domain (IPR008977), were included among the Copper type II OrtFams.

We next considered nemS-cIntFams that have supporting evidence for expression from core nematode species. The *A. suum* intestine offers the most detail in this regard. Relative overexpression in intestinal compared to other adult worm tissues (8 genes (Rosa et al., 2014)) provided one filter, and the next filter was direct detection of the predicted protein in intestinal tissue by mass spectrometry (12 proteins (Rosa et al., 2015)) (Table S7). This process identified two nemS-IntFams of interest: (i) OrtFam_1531, representing nidogen domain-containing proteins, and including F54D1.6 (*C. elegans*), which has lysosome and sub-cellular localization variants (Winter et al., 2012) that may implicate endocytic functions at the apical intestinal membrane and (ii) OrtFam_1264, representing N-acyltransferase proteins. Some acyltransferase proteins in nematodes have been previously shown to modify the surface of the nematode hindgut, affecting general surface integrity as well as bacterial pathogen adhesion (Gravato-Nobre and Hodgkin, 2008). Collectively, the nemS-cIntFams identify a restricted set of recognizable, basic functions that have acquired molecular characteristics that distinguish nematodes from other outgroup species. These nemS-cIntFams provide specific examples of, and means to investigate, functions postulated to have central roles in the evolutionary success of the Nematoda.

Next, we sought to identify functions that may have contributed to the success of different nematode clades. For instance, functional enrichment categories from csc-IntFams of clade I nematodes resolved serine peptidases as important for the success of the three species analyzed in this clade. While no enriched functions were identified specifically for clade III nematodes, zinc finger domains (inclusive of nuclear hormone receptors, NHRs), which were especially well documented, and oxygen binding (globin) functions were identified as important for the success of nematodes in both clades III and V. NHR functions were independently identified as enriched for clade V nematodes along with carbohydrate binding (galectin). These results provide important information on functions that appear to have played roles central to the evolution of the lineages discussed and those further outlined in Table S6. Oxygen binding, as related to nematode globins, purportedly function to scavenge oxygen in pseudocoelomic fluid and thus maintain low homeostatic oxygen levels (Minning et al., 1999). It will be important to determine if expression in the intestine indicates a similar function in intestinal cells, or if intestinal cells synthesize and secrete globins into the pseudocoelom, or both. The emphasis resolved on NHR and zinc binding proteins highlights the general importance of families of these proteins in the evolution of the Nematoda (described below).

**Fig. 4:**
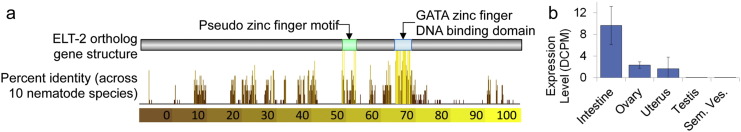
Conservation and expression level of the ELT-2 gene. (a) ELT-2 like protein domain architecture and its overall sequence conservation from the 10 nematode species. (b) Expression profile of the ELT-2 like gene in *A. suum* (*GS_05212*).

On inspection of 1246 csc-IntFam annotations, we also found numerous IPR domains that were shared among csc-IntFams across the different clades (Figs. 3; S6). This similarity could indicate that proteins with functions ancestral to the Nematoda have diverged to represent lineage-specific functions, while retaining functions of ancestral importance (Table S6). One example that supports this possibility derives from zinc binding proteins, of which numerous distinct subfamilies are represented among clades I, III, V and III & V. One subgroup is NHR/GATA transcription factors (Table S6), which include ELT-2, a major intestinal gene transcription factor in *C. elegans* (McGhee et al., 2009). Unexpectedly, ELT-2 orthologs were not detected in nemS-IntFams, despite detection of homologous proteins for each of the nematode species investigated (Figs. 4; S7). In each case, intestinal ELT-2 homologs from core species displayed high identity in the zinc finger-DNA binding region along with the upstream pseudo-zinc finger domain. The same observation was made for best match ELT-2-like proteins from non-core nematode species. The *H. contortus* example functions as an ELT-2 transcription factor in *C. elegans* intestinal cells (Couthier et al., 2004), despite highly divergent protein sequences outside the two zinc finger homology regions. Therefore, as a specific example of a zinc binding protein, ELT-2-like proteins are conserved in a pan-Nematoda context and appear to have had a central role in the evolution of the Nematoda, but lack sufficient amino acid sequence to be resolved as nem- or nemS-cIntFams. This observation is important, as it relates to an evolutionary process that may have shaped the success of the Nematoda, and clarifies efforts that may be needed to identify key factors of that success. The implied process would also be distinct from one involving convergent evolution.

This analysis of functions attributable to IntFams identified a wide range of IntFams and associated functions with significance at the pan-phylum and/or lineage levels for nematodes. This broad significance is conveyed in part by the natural history of IntFams, which is annotated in the cIntFam and its subgroupings, and then in csc-IntFams (Table S3). IntFam members comprising each of the cIntFam and its subgroups, along with csc-IntFams, can now be used to determine the potential application of these research results among members of the phylum.

#### 3.4.2 Species-specific Intestinal Proteins and Associated Functions

sIntPros (Group d) and ss-IntFams (Fig. 2, Group l), in combination, identify intestinal proteins that are specific to each of the core species and may reflect adaptations of importance for the success of each species (or possibly the lineages within a clade leading to that species). The total number of proteins in these two groups is 1739, 3236, and 2803 for the three core species (*T. suis*, *A. suum*, and *H. contortus*, respectively). It is notable that the 1739 proteins identified for *T. suis* reflect differences with the sister species *T. muris*. Consequently, the species-specific proteins identified here reflect a large potential for contributing diversity among nematode species even within genera.

Similar to our approach with csc-IntFams, we explored the possibility that species-specific intestinal proteins include interspecies homologs that could reflect divergence of otherwise conserved functions, or convergent acquisition of functions. To simplify comparisons, we focused on the 5775 singleton intestinal proteins, sIntPro, for each of the core species, of which only 430 (7.4%, 430/5775) shared homology with a protein(s) from one or more of the other core species (Fig. 2, Group d; Fig. S6E). Although functions of many of the proteins remain unassignable (3740) by available annotation methods, there were 2035 proteins from all three species annotated with 1439 unique IPR domains. 477 of the 1439 unique IPR domains were found to be in common with proteins from two or three of the core species (Fig. S6E). Thus, functional similarity was evident among intestinal proteins otherwise characterized as species-specific (Table S6). Divergence and adaptation of an ancestral function(s) to meet lineage or species-specific needs might explain this similarity, much like the proposal for ELT-2 proteins. Concurrently, a high percentage of proteins with intestinal transcript support in core species showed no indication of functional similarity across these species, again reflecting a large source of diversity among nematode species.

### 3.5 IntFams as Candidates for Treatment and Control of Parasitic Nematode Infections

IntFam protein functions that are conserved among members of multiple nematode lineages have potential to serve as targets for new broad-spectrum therapies for treatment and parasite control. Proteins that have potential as drug targets include inter-phylum, taxonomically conserved, and highly homologous proteins that have sufficiently diverged in the parasite to enable selective targeting and avoidance of drug toxicity to the host. To this end, we focused on insertions and deletions (indels) in parasite proteins relative to host-group proteins, because indels generally have a greater effect on protein structure and function than single amino acid changes that result from substitutions (Hormozdiari et al., 2009; Salari et al., 2008). Indels can also create unique ligand binding sites on the protein surface (Studer et al., 2013).

The analysis of indels takes into account proteins from all 10,772 nematode IntFams (54,469) and host outgroup species (30,804) included in this investigation. In this context, nematode proteins from 5551 IntFams have indels, of which 5331 IntFams have nematode-specific indels, including 852 of the 863 uni-cIntFams. Overall, the number of nematode-specific deletions was higher than the number of insertions, and the deletion length was significantly longer (p < 1 × 10^− 10^; T-test; Table 2), confirming (Wang et al., 2009) and substantially expanding on previous findings based on partial genomes. Next, the location of nematode-specific indels were mapped according to structure of the homologous proteins from the protein database (PDB), the content of which restricted the number of nematode proteins that could be analyzed. Nevertheless, 15,577 nematode proteins (2582 IntFams) have PDB homologs of which 838 nematode proteins (508 IntFams) have indels close to potential ligand binding sites, among which 270 proteins originated from at least one of the three core species (236 uni-cIntFams). Hence, we identified broadly conserved nematode proteins with molecular differences compared to host proteins that warrant further studies as drug-target candidates. This predictive application will improve along with expansion of content in protein structural databases. The results are also likely to reflect differences in protein functions between parasite and hosts. All the IntFam alignments and locations of the identified indels are available at Nematode.net (Martin et al., 2015).

**Fig. 5:**
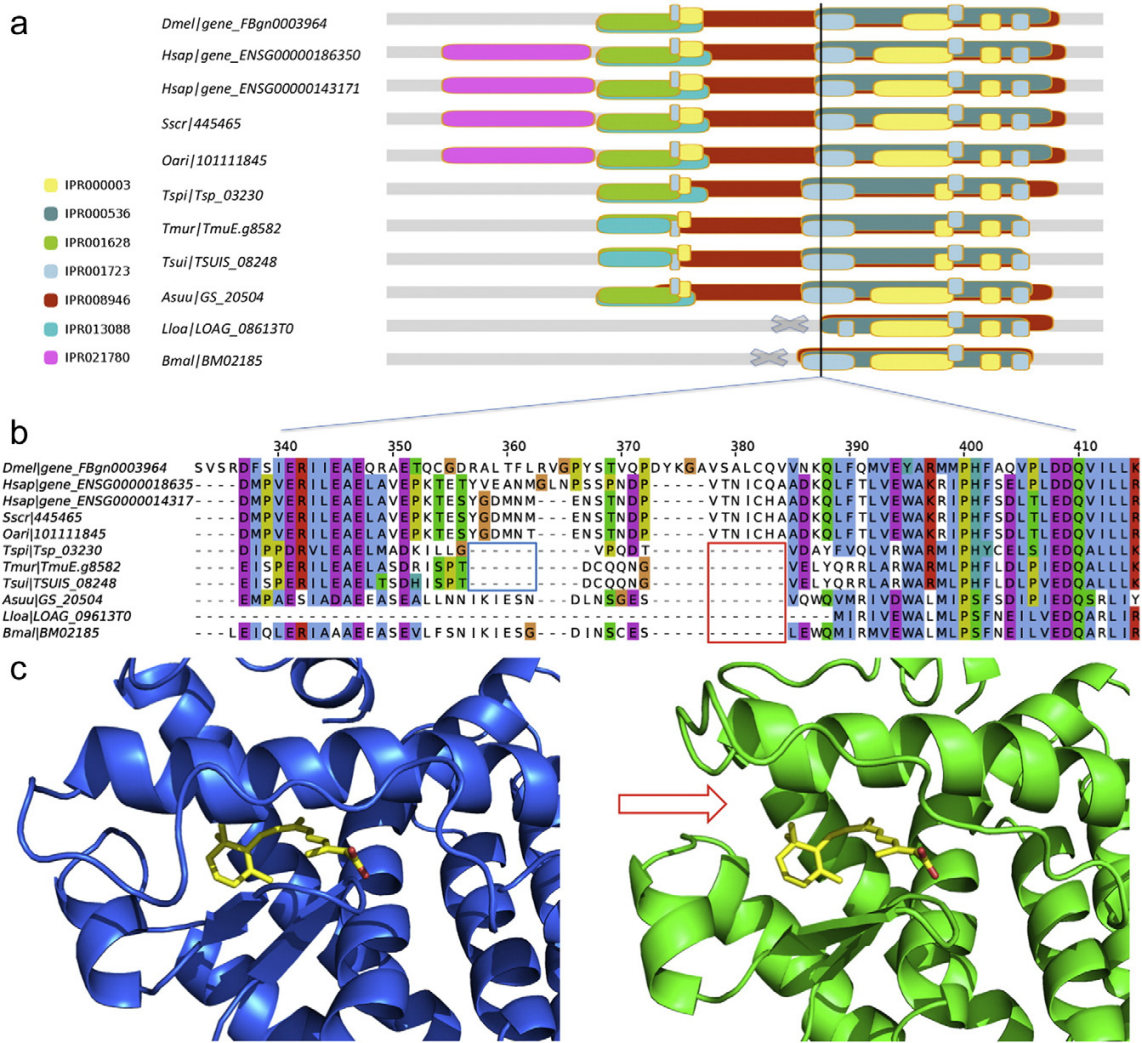
A 7 amino acid deletion in retinoid X receptor α (RXR-α) of *A. suum* creates a unique ligand binding site. (a) IPR domain annotations for the orthologs within the IntFam. (b) Sequence alignment of the deleted region in RXR-α from *A. suum* with all the other members of the intestinal protein family. The deleted region is in red box. Another deletion specific to clade I is in blue box. (c) Side-by-side view of the crystal structure for *H. sapiens* (PDB ID: 3E00) with the homology model of *A. suum*. Crystal structure is colored in blue; model is colored in green. The natural ligand 9-cis retinoic acid is shown in yellow stick model in both panels. The cavity created by the deletion is pointed out by a red arrow in the model.

The *A. suum* protein GS_20504 (OrtFam_6311, member of ncIntFam) provides an example for how parasite specific indels might be explored for selective drug targeting. This protein is an ortholog of human retinoid X receptor α (RXR-α) and a member of the steroid/thyroid hormone superfamily of nuclear hormone receptors (NHRs). Members of this NHR superfamily mainly function as transcription factors with roles in development, cell differentiation, metabolism, and cell death. The human ortholog in this case has been studied as a drug target of a wide variety of diseases from cancer, dementia to diabetes (Dawson and Xia, 2012). We identified a 7 AA-long deletion near the binding site of the natural ligand 9-cis-retinoic acid in the ligand-binding domain (LBD) of RXR-α. When compared with the homologous structure from humans (Fig. 5a), the deletion creates a cavity within which selective inhibitors might be designed to bind at and block the natural ligand. The sequence alignment for all members in the IntFam also shows that the gap is shared among orthologous proteins from six nematode species, suggesting that a drug based on this concept could also have broad spectrum application (Fig. 5b).

These analyses identified a large number of IntFams with indels that are specific to nematodes in comparison to mammalian hosts. These indels have high potential for therapeutic targeting that is specific to the parasite. The full potential of this pan-Nematoda database of IntFam indels remains unrealized due to limitations on content of protein-structure databases. Nevertheless, the depth, breadth and phylogenetic application of content make the indel database a valuable resource for both elucidating nematode protein functions and methods to inhibit them.

## 4 Discussion

Biological characteristics unique, by comparison to other tissues, to the intestine of parasitic nematodes have led to promising new approaches for therapies to treat and control infections caused by these pathogens of humans, animals and possibly plants (Jasmer et al., 1993, 2000, 2007; Smith, 1993; Hu et al., 2010, 2012; Kao et al., 2011). Despite the promising advances, research on the intestine has been impeded by a poor understanding of the protein constituents that comprise the intestinal proteome and perform functions essential for survival of nematodes. While it is expected that many intestinal cell characteristics will be conserved among nematodes, expectations of high functional diversity among the many and varied nematode species are raised simply by considering the broad diversity in intestinal morphology and trophic niches inhabited by nematodes. Until now, viewpoints on relative conservation and diversity of intestinal cell proteins among species have been difficult to generate. Research presented here has made a major step in establishing quantitative measures on conservation and diversity of intestinal functions and in a pan-Nematoda context. These advances led to establishment of an extensive database that pinpoints protein sites with application towards development of new therapies against many pathogenic nematodes.

Pan-Nematoda or lineage-specific conservation of intestinal cell functions was documented at several phylogenetic levels, identifying IntFams and related functions that have significance for success of many or all nematodes contained in each of the phylogenetic groups investigated. This level of resolution at the level of a specific nematode organ is unique and will facilitate immediate, and informed, research applications at many phylogenetic levels to address problems related to parasitic nematodes of humans (e.g. hookworms, threadworms, ascarids, filarids, and whipworms) and/or animals (e.g. strongyles, ascarids, filarids, and whipworms), many of which due to their size will not meaningfully support this kind of research on intestinal cell functions. We predict similar application to plant parasitic nematodes.

Data presented outline specific intestinal cell functions (inclusive of immunoglobulin domains, oxygen binding and oxidoreductive activities, endocytic transcriptional process, to name a few) that can now be investigated to determine the importance of these functions for survival of species in the lineages designated. Clarification of functions with this significance in the comprehensive manner has not been previously accomplished and reported for any nematode tissue or organ. Biological characteristics of the core species investigated that were pertinent to reaching this goal included phylogenetic representation (clades I, III, V), biological diversity (trophic niche, tissue migration and life cycle), and support for tissue dissection. The results provide clear support for application of this approach to other tissues, which when coupled with this research will clarify with high resolution pan-Nematoda genomic functions as related gene and protein expression in adult nematodes.

The extensive information on the natural history of IntFam births/deaths and family members across the phylogenetic diversity of the Nematoda provides a new prospective tool to navigate potential application of research before engaging it, which broadly extends to research on species of importance to biomedical, agricultural and general veterinary sciences.

The variation in IntFam presence and members of IntFams among phyla, nematode clades and nematode species reflects tremendous diversity that may underlie diversity observed across the Nematoda as related to intestinal morphology and trophic niches occupied by nematode species. Collective information provides a platform to elucidate conserved and diverse features of intestinal cell physiology among nematodes, which has basic value towards therapeutic targeting of the intestine for treatment and control of parasitic nematode infections. Concurrently, functions that lack pan-Nematoda ortholog status, such as ELT-2-like transcription factors, other zinc binding proteins, other csc-IntFam proteins, and even species-specific proteins, may reflect ancestral functions that have diverged beyond recognition as such. Hence, along with the conserved IntFams identified here, additional intestinal cell functions, which lack ortholog status by our methods, are likely to exist as pan-Nematoda conserved intestinal cell functions. This understanding coupled with capacity established in this research will support efforts to delineate those functions.

Indel analysis provided an effective approach to explore the potential for therapeutic targeting of broadly conserved intestinal cell functions in parasitic nematodes. The results clearly resolved this potential at the molecular level, which extended to nematode proteins that retain relatively high homology with proteins from mammals. Hence, practical application of the results may extend to pathogen proteins with even high homology to host proteins. These findings coupled with the established databases could figure significantly in systematic approaches to identify specific intestinal cell targets for therapeutic applications.

### 4.1 Data Availability

The RNAseq data generated in this study are available at NCBI under the SRA accession numbers for *T. suis* SRX736470 and SRX736471; for *A. suum* SRX278152, SRX278166, SRX278128, SRX278125 and for *H. contortus* SRX736496 and SRX736495. The expression profiles of the intestinal genes and the multiple alignments along with indel locations are available for download at Nematode.net (http://nematode.net).

The following are the supplementary data related to this article.

Supplementary material

Table S2 Protein counts and IDs per species for every OrtFam (31,014).

Table S3 Annotation and protein counts for every IntFam (10,772).

Table S6 Molecular Functions (GO) terms and Domains (IPR) significantly enriched among intestinal groups and associated with proteins in Fig. 2 groups d and m.

Table S7 Annotation and expression data for *A. suum* nemS-cIntFam genes.

## Author Contributions

M.M. and D.P.J. jointly conceived, designed and supervised the research. D.P.J. performed the experiments and provided the material. Q.W. and B.A.R. analyzed the data and prepared the figures and tables. Q.W., B.A.R., D.P.J. and M.M. wrote the manuscript.

## Figures and Tables

The data were obtained from Rosa et al. (2014).

**Table 1:** RNA-seq mapping results and intestinal protein families (IntFams) detected and inferred for the three species.

Species	Library	Reads (million pairs)		Total hits (millions)	Intestine-expressed genes			Unique protein families^a^	Inferred by orthology^b^		Union of protein families (IntFam)
Total	Clean	Per sample	Per species	In protein families	Total Intfam	Total genes
*T. suis*	Male	70.6	68.3	69.9	7745	7898	6345	5680	5833	6709	10,772
Female	83.2	81.3	88.7	7096
*A. suum*	Male1	29.2	27.3	36.0	8918	11,109	8115	7089	7666	9267
Female1	31.3	28.9	32.3	10,280
Male2	29.3	28.0	36.8	7411
Female2	22.0	15.2	19.3	8958
*H. contortus*	Male	83.7	71.3	62.1	9590	10,824	9596	6386	7598	13,996
Female	100.7	93.1	106.8	8735
						29,831	24,056	19,155	21,097	29,972

a Fig. S3a.

b Fig. S3b.

**Table 2:** Characteristics of nematode-specific insertions and deletions identified in the intestinal protein families^a^.

Size bin (AA)	Intestine (5331 IntFam)			
Count		Percentage	
Deletions	Insertions	Deletions	Insertions
0–4	17,347	16,503	40.9%	54.9%
5–10	7178	5667	16.9%	18.9%
11–19	5255	3720	12.4%	12.4%
20–200	12,589	4160	29.7%	13.8%
Total	42,369	30,050	100%	100%
Average	20.22	9.76	–	–
SD	30.94	15.93	–	–
Sizeable/family	4.69	2.54	–	–
Organisms studied	10 nematodes	5 references	–	–

a All multiple sequence alignments and locations of the identified indels are available at http://nematode.net.
