# Supplementary material for: Pan-Nematoda Transcriptomic Elucidation of Essential Intestinal Functions and Therapeutic Targets With Broad Potential
Source: eBioMedicine. 2015 Jul 29;2(9):1079–89. doi: 10.1016/j.ebiom.2015.07.030 (PMC4587998; doi:10.1016/j.ebiom.2015.07.030)
Supplement: Supplementary file 1 — Supplementary material [file mmc1.docx]

# Supplementary Figures and Tables

# 1-A. Figures


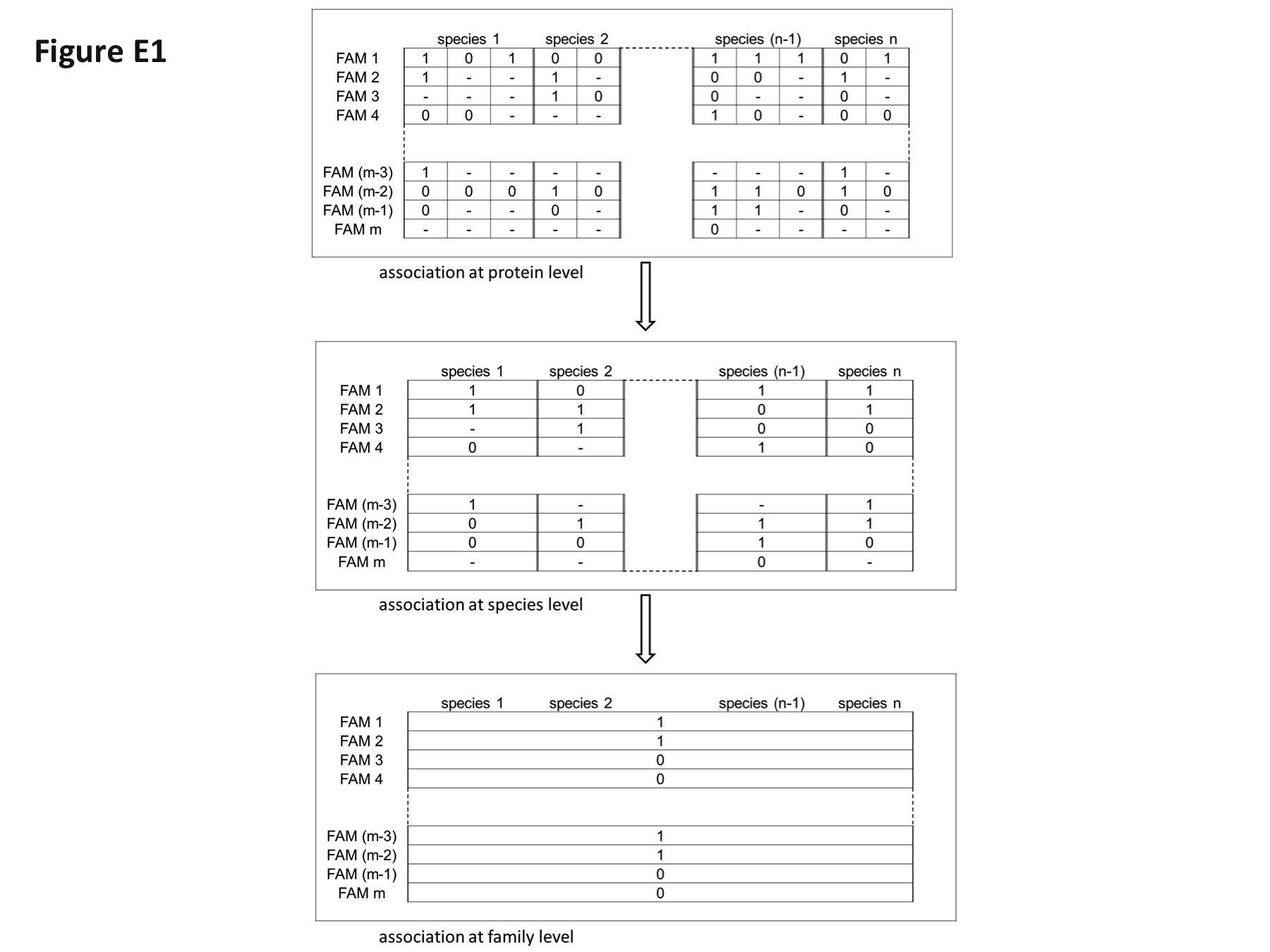


**Figure S1: The stepwise approach to identify a functional term association within a protein family.**

The process depicted represents the process of associating (1) or not associating (0) a single functional term (GO term or IPR domain) to each orthologous protein family (FAM). In the first panel, a matrix is produced in which proteins are associated with the term (1), not associated (0) or not present in the FAM (-). In the second panel, if 50% or more of the FAM proteins within a species are associated with the term, then for that FAM, the species is associated (1) with the term. Otherwise, the FAM may not be associated with the term (0) or it may not be present within a given species (-). Finally, for a given FAM, if more than 50% of the species are associated with the term, then the FAM is associated with the term (1); Otherwise, it is not (0).


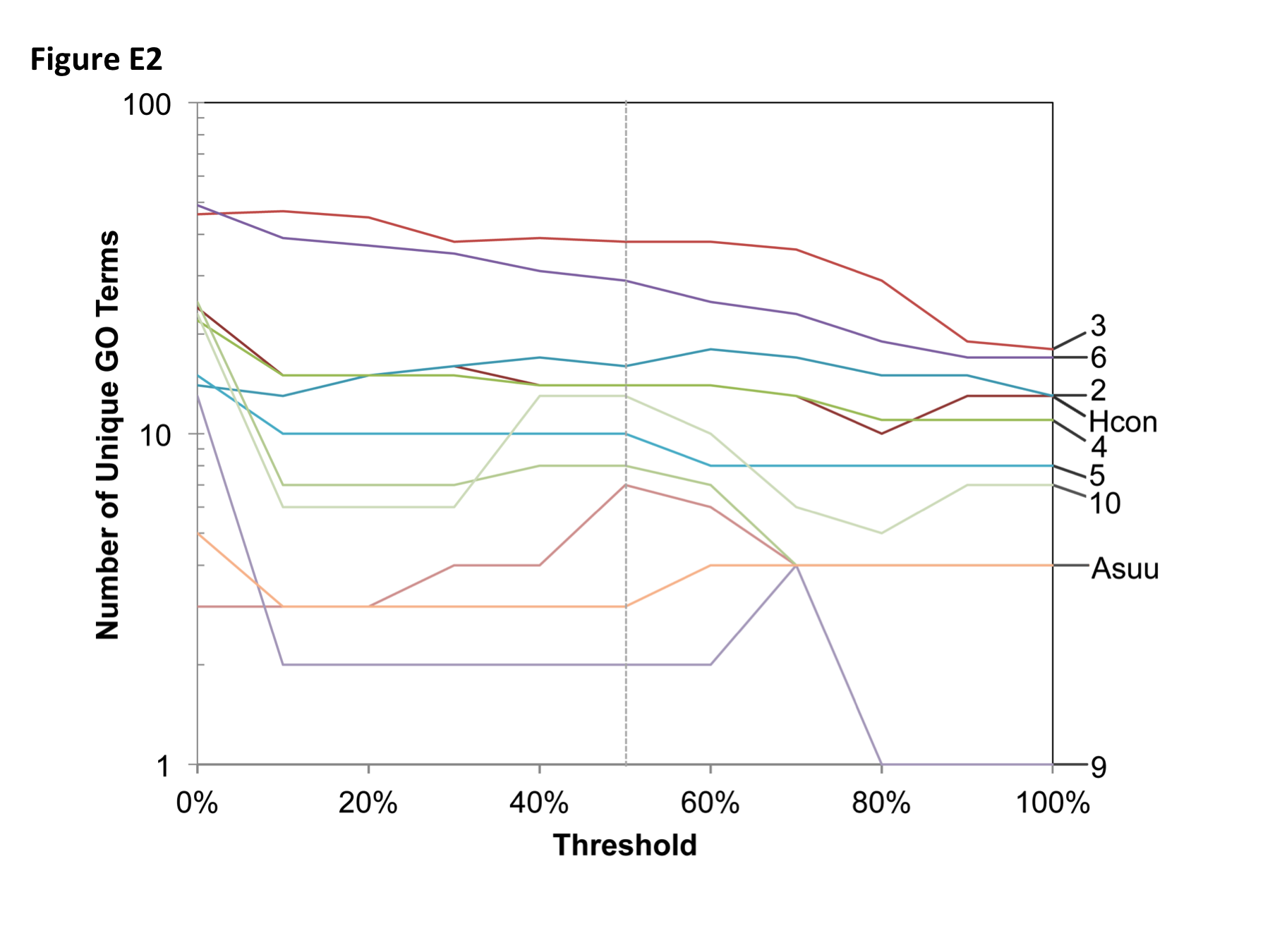


**Figure S2: The number of statistically different GO terms (p < 0.05) in the gained intestinal protein families compared with the background (all IntFam at that lineage) derived at different cutoffs in the stepwise approach (Figure 2). Each lineage with non-zero GO terms was labeled with different legends.**


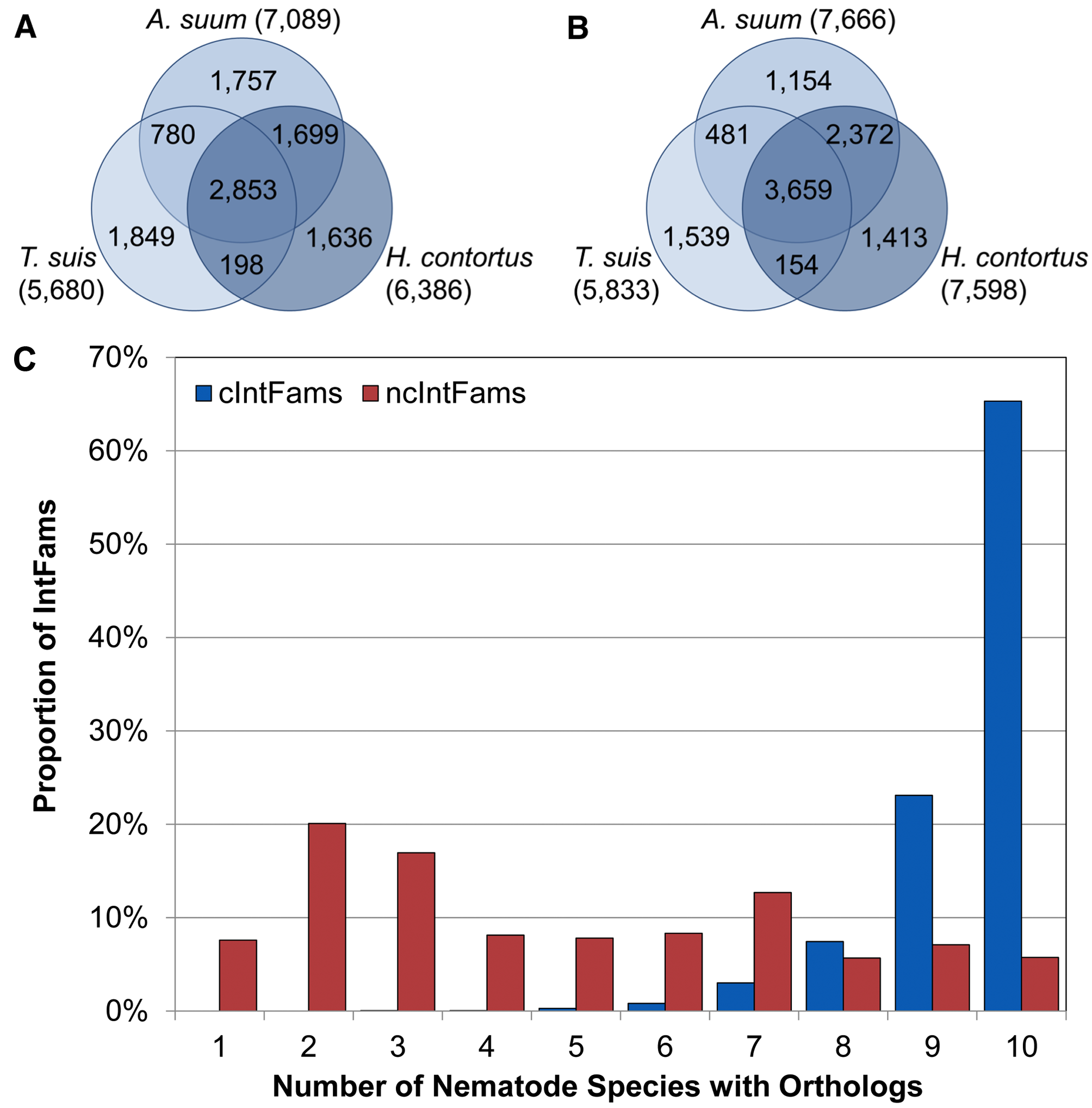


**Figure S3: Adult intestine protein family categorization.**

(A) The protein families were identified directly from RNA-Seq data (without inference). (B) Protein families inferred from the transcript evidence of the other two species. (C) The total number of nematode species represented among cIntFams and ncIntFams.


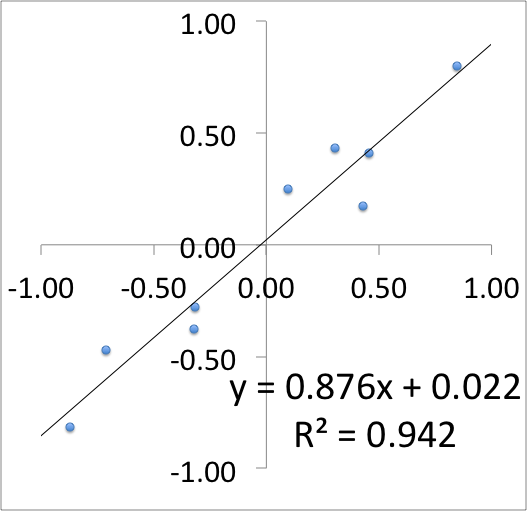


**Figure S4: Correlation (r^2^=0.942; p=1.5e-4) between the PFCI for IntFams at nodes within the Nematoda and the PFCI for whole proteomes.**


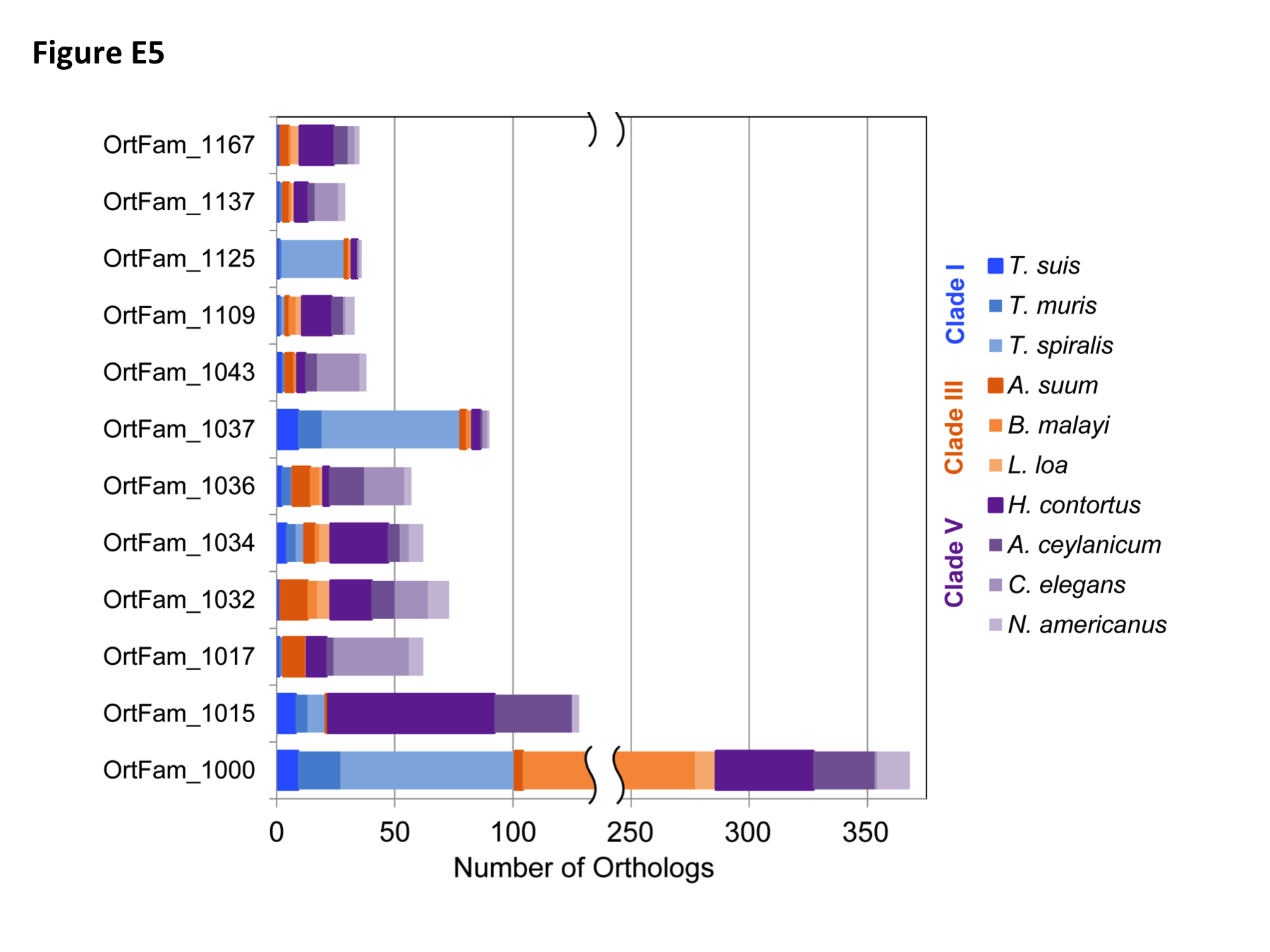


**Figure S5: Number of orthologous proteins within the 12 IntFams with 10 fold or higher expansion in Nematoda.**


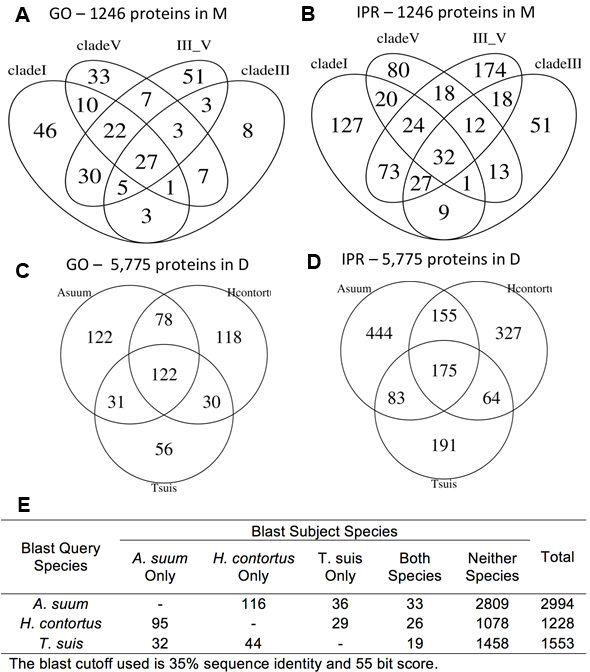


**Figure S6: Overlapping functional terms associated with subsets of genes.**

(A) Gene Ontology (GO) terms associated with csc-IntFams. (B) InterPro (IPR) domains associated with csc-IntFams. (C) Gene Ontology (GO) terms associated with singletons encoded by genes expressed in the intestine. (D) InterPro (IPR) domains associated with singletons encoded by genes expressed in the intestine. (E) Homology shared between the singleton proteins (Figure 2, group D) of the core species.


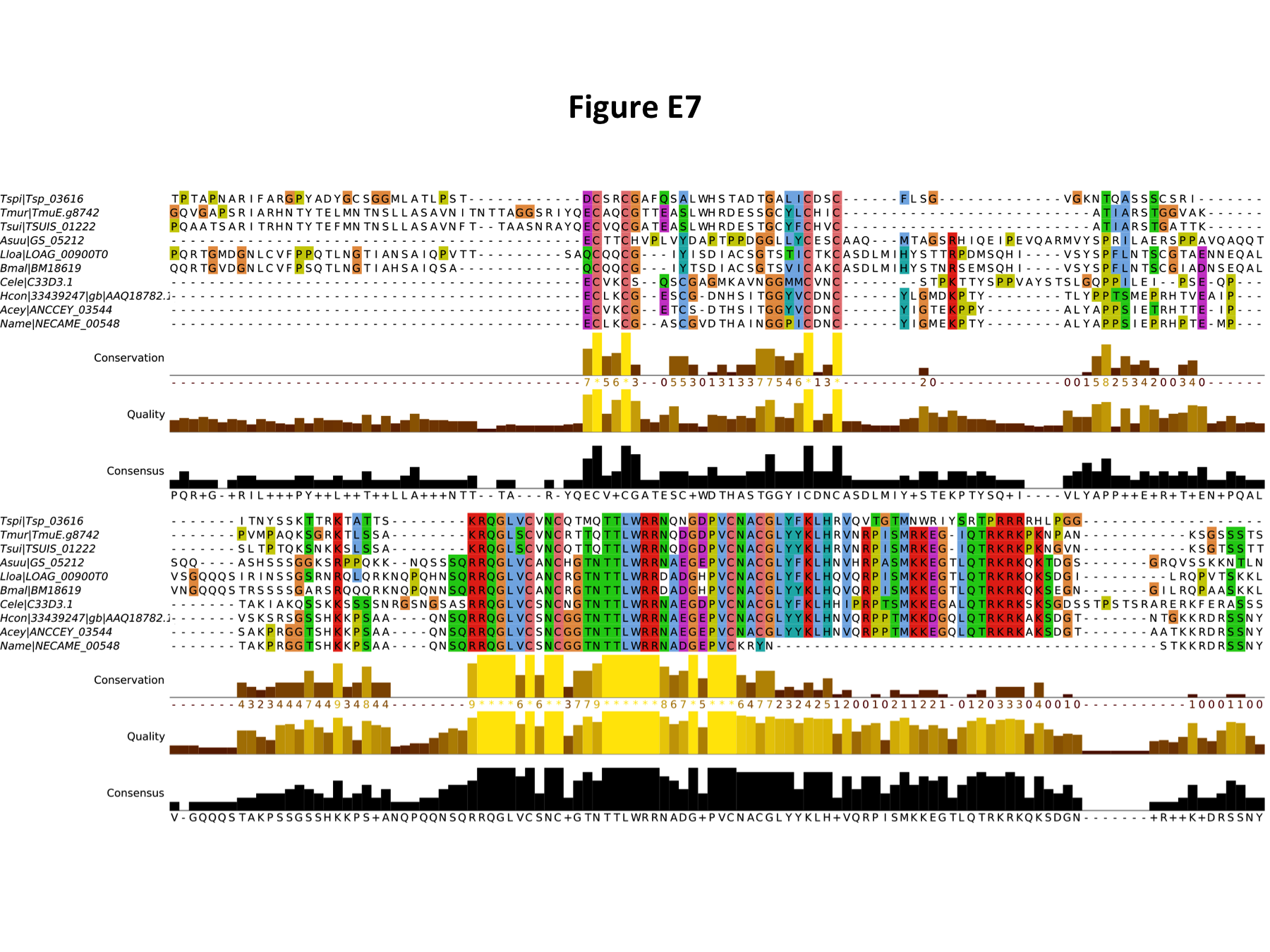


**Figure S7: Sequence alignments of the ELT-2 like proteins. Only sequence regions that include the two conserved domains (psudo zinc finger motif and GATA zinc finger DNA binding domain) are shown.**

**1-B. Tables**

| **Table S1, Related to Figure 2: Protein, protein family and singleton counts for proteomes used in this study.** | | | | | | |
| --- | --- | --- | --- | --- | --- | --- |
| Species | | Abbre-viation | Proteins in proteome | Proteins in family | Protein families | Singletons |
| Clade I Nematodes | ***T. suis*** | Tsui | 9,841 | 7,371 | 6,435 | **2,470** |
|  | *T. muris* | Tmur | 9,403 | 7,738 | 6,380 | 1,665 |
|  | *T. spiralis* | Tspi | 15,840 | 10,936 | 6,244 | 4,904 |
| Clade III Nematodes | ***A. suum*** | Asuu | 18,542 | 11,110 | 9,256 | **7,432** |
|  | *B. malayi* | Bmal | 18,348 | 13,243 | 8,529 | 5,105 |
|  | *L. loa* | Lloa | 14,893 | 10,378 | 8,915 | 4,515 |
| Clade V Nematodes | ***H. contortus*** | Hcon | 21,903 | 18,511 | 10,255 | **3,392** |
|  | *A. ceylanicum* | Acey | 15,919 | 12,033 | 9,450 | 3,886 |
|  | *C. elegans* | Cele | 20,517 | 16,766 | 9,913 | 3,751 |
|  | *N. americanus* | Name | 19,151 | 12,752 | 10,333 | 6,399 |
| Hosts | *H. sapiens* | Hsap | 22,400 | 20,726 | 14,737 | 1,674 |
|  | *O. aries* | Oari | 19,108 | 18,090 | 14,567 | 1,018 |
|  | *S. scrofa* | Sscr | 22,001 | 18,605 | 13,894 | 3,396 |
| Outgroups | *D. melanogaster* | Dmel | 13,917 | 10,170 | 6,634 | 3,747 |
|  | *S. cerevisiae* | Scer | 6,692 | 4,017 | 2,761 | 2,675 |
| Total | |  | 248,475 | 192,446 | 31,014 | 56,029 |
| **Bold**, core species. | |  |  |  |  |  |

**Table S2, related to Figure 2: Protein counts and IDs per species for every OrtFam (31,014).**

(See external excel file, Table S2.xlsx)

**Table S3, related to Figure 2: Annotation and protein counts for every IntFam (10,772),.**

(See external excel file, Table S3.xlsx)

| **Table S4, related to Figure 3A and B. Protein Family birth and death throughout nematode evolution.** | | | | | | | |
| --- | --- | --- | --- | --- | --- | --- | --- |
| Node | Previous node | All protein families from proteome | | | Intestinal protein families (transcriptome) | | |
|  |  | birth (PFb) | death (PFd) | PFCI=log (PFb/PFd) | birth (PFb) | death (PFd) | PFCI=log (PFb/PFd) |
| 1 | root | 2406 | - | - | 2080 | - | - |
| *S. cerevisiae* | 1 | 355 | - | - | - | - | - |
| 2 | 1 | 4112 | - | - | 3431 | - | - |
| 12 | 2 | 873 | 88 | 1.00 | - | 31 | - |
| 3 | 2 | 1002 | 202 | 0.70 | 755 | - | - |
| 4 | 3 | 691 | 1435 | -0.32 | 483 | 916 | -0.28 |
| 6 | 3 | 2883 | 409 | 0.85 | 1671 | 265 | 0.80 |
| 5 | 4 | 850 | 421 | 0.31 | 596 | 221 | 0.43 |
| *T. spiralis* | 4 | 850 | 1180 | -0.14 | - | 1042 | - |
| *T. muris* | 5 | 125 | 748 | -0.78 | - | 667 | - |
| *T. suis* | 5 | 88 | 656 | -0.87 | 68 | 443 | -0.81 |
| 7 | 6 | 730 | 583 | 0.10 | 418 | 236 | 0.25 |
| 9 | 6 | 1161 | 434 | 0.43 | 376 | 252 | 0.17 |
| 8 | 7 | 858 | 1558 | -0.26 | - | 1077 | - |
| *A. suum* | 7 | 164 | 847 | -0.71 | 96 | 284 | -0.47 |
| *B. malayi* | 8 | 526 | 1236 | -0.37 | - | 803 | - |
| *L .loa* | 8 | 171 | 495 | -0.46 | - | 231 | - |
| 10 | 9 | 1009 | 354 | 0.45 | 454 | 177 | 0.41 |
| *C. elegans* | 9 | 867 | 1473 | -0.23 | - | 764 | - |
| 11 | 10 | 582 | 572 | 0.01 | - | 346 | - |
| *H. contortus* | 10 | 840 | 1759 | -0.32 | 344 | 819 | -0.38 |
| *A. ceylanicum* | 11 | 298 | 2032 | -0.83 | - | 1234 | - |
| *N. americanus* | 11 | 266 | 1117 | -0.62 | - | 532 | - |
| 13 | 12 | 7843 | 431 | 1.26 | - | 297 | - |
| *D. melanogaster* | 12 | 583 | 1252 | -0.33 | - | 796 | - |
| 14 | 13 | 436 | 54 | 0.91 | - | 21 | - |
| *H. sapiens* | 13 | 201 | 179 | 0.05 | - | 106 | - |
| *O. aries* | 14 | 63 | 593 | -0.97 | - | 113 | - |
| *S. scrofa* | 14 | 181 | 1384 | -0.88 | - | 275 | - |

**Table S5, related to Figure 3C. Gene gain and loss events in the universal families (UniFams). The events were normalized to both the branch lengths and the total number of universal families in Figure 3. Refer to Figure 3 for the lineage denotations.**

| Node | Previous node | Branch length | Universal families with duplicates | | Universal families without duplicates | | Universal families (total) | | Normalized by branch length (per family) | |
| --- | --- | --- | --- | --- | --- | --- | --- | --- | --- | --- |
|  |  |  | Duplication | Loss | Duplication | Loss | Duplication | Loss | Duplication | Loss |
| 1 | root |  | 87 | 0 | 0 | 0 | 87 | 0 |  |  |
| *S. cerevisiae* | 1 | 0.80 | 181 | 64 | 0 | 0 | 181 | 64 | 0.26 | 0.09 |
| 2 | 1 | 0.15 | 1061 | 2 | 123 | 0 | 1184 | 2 | 9.07 | 0.02 |
| 3 | 2 | 0.03 | 133 | 411 | 3 | 39 | 136 | 450 | 5.37 | 17.76 |
| 4 | 3 | 0.21 | 57 | 672 | 0 | 73 | 57 | 745 | 0.31 | 4.04 |
| *T. spiralis* | 4 | 0.40 | 101 | 177 | 0 | 14 | 101 | 191 | 0.29 | 0.55 |
| 5 | 4 | 0.29 | 23 | 104 | 0 | 14 | 23 | 118 | 0.09 | 0.48 |
| *T. suis* | 5 | 0.12 | 15 | 57 | 0 | 0 | 15 | 57 | 0.15 | 0.57 |
| *T. muris* | 5 | 0.12 | 15 | 84 | 0 | 0 | 15 | 84 | 0.15 | 0.84 |
| 6 | 3 | 0.21 | 433 | 465 | 21 | 79 | 454 | 544 | 2.45 | 2.94 |
| 7 | 6 | 0.17 | 46 | 457 | 6 | 21 | 52 | 478 | 0.35 | 3.22 |
| *A. suum* | 7 | 0.23 | 38 | 224 | 0 | 14 | 38 | 238 | 0.19 | 1.21 |
| 8 | 7 | 0.17 | 42 | 253 | 0 | 8 | 42 | 261 | 0.28 | 1.76 |
| *L. loa* | 8 | 0.06 | 1 | 84 | 0 | 6 | 1 | 90 | 0.02 | 1.83 |
| *B. malayi* | 8 | 0.06 | 58 | 88 | 0 | 6 | 58 | 94 | 1.18 | 1.91 |
| 9 | 6 | 0.13 | 104 | 331 | 3 | 12 | 107 | 343 | 0.99 | 3.16 |
| *C. elegans* | 9 | 0.28 | 86 | 461 | 0 | 20 | 86 | 481 | 0.36 | 2.03 |
| 10 | 9 | 0.16 | 335 | 202 | 39 | 12 | 374 | 214 | 2.65 | 1.51 |
| *H. contortus* | 10 | 0.11 | 357 | 519 | 0 | 47 | 357 | 566 | 3.71 | 5.88 |
| 11 | 10 | 0.04 | 10 | 150 | 0 | 4 | 10 | 154 | 0.28 | 4.24 |
| *N. americanus* | 11 | 0.07 | 9 | 391 | 0 | 43 | 9 | 434 | 0.15 | 7.25 |
| *A. ceylanicum* | 11 | 0.07 | 39 | 408 | 0 | 43 | 39 | 451 | 0.65 | 7.53 |
| 12 | 2 | 0.20 | 38 | 685 | 0 | 81 | 38 | 766 | 0.22 | 4.44 |
| *D. melanogaster* | 12 | 0.44 | 191 | 418 | 0 | 42 | 191 | 460 | 0.50 | 1.20 |
| 13 | 12 | 0.40 | 1051 | 349 | 69 | 42 | 1120 | 391 | 3.21 | 1.12 |
| *H. sapiens* | 13 | 0.04 | 141 | 702 | 0 | 69 | 141 | 771 | 4.02 | 21.97 |
| 14 | 13 | 0.01 | 19 | 89 | 0 | 0 | 19 | 89 | 3.51 | 16.42 |
| *S. scrofa* | 14 | 0.03 | 57 | 672 | 0 | 69 | 57 | 741 | 1.92 | 24.97 |
| *O. aries* | 14 | 0.03 | 39 | 659 | 0 | 69 | 39 | 728 | 1.31 | 24.53 |
| Total |  |  | 4767 | 9178 | 264 | 827 | 5031 | 10005 |  |  |
| Total in Nematoda |  |  | 1769 | 5127 | 69 | 416 | 1838 | 5543 |  |  |

**Table S6, related to discussion (intestinal cell functions): Molecular Functions (GO) terms and Domains (IPR) significantly enriched among intestinal groups and associated with proteins in Fig 2 Groups D and M.**

(See external excel file, Table S6.xlsx)

**Table S7, related to discussion (intestinal cell functions): Annotation and expression data for *A. suum* nemS-cIntFam genes**

(See external excel file, Table S7.xlsx)

# 2. Supplementary Materials and Methods

**Data collection.** Whole deduced proteome data (hereafter refer to as ‘proteomes’) from 15 eukaryotic species (10 nematodes and 5 hosts/outgroups) were downloaded as follows: The outgroups *Homo sapiens, Saccharomyces cerevisiae* and *Drosophila melanogaster* were from Ensembl ([Kinsella et al., 2011](#_ENREF_13)) release 67, and *Sus scrofa* and *Ovis aries* were from GenBank ([Benson et al., 2012](#_ENREF_1)) release 103 and 100 (respectively). For the nematodes, *Caenorhabditis elegans* and *Brugia malayi* were from Wormbase ([Harris et al., 2013](#_ENREF_8)) WS230, and *Trichinella spiralis, Ascaris suum, Haemonchus contortus* and *Necator Americanus* were from published data ([Jex et al., 2011](#_ENREF_12); [Laing et al., 2013](#_ENREF_14); [Mitreva et al., 2011](#_ENREF_16); [Tang et al., 2014](#_ENREF_19)). *Trichuris muris* was from Sanger Institute (<ftp://ftp.sanger.ac.uk/pub/pathogens/Trichuris/muris/>). *Loa loa* was from Broad Institute ([Desjardins et al., 2013](#_ENREF_3)) (<http://www.broadinstitute.org>). The other 2 nematode species, *Ancylostoma ceylanicum* and *Trichuris suis* were from our in-house sequencing data. When gene loci were represented by multiple isoforms, only the longest one was included in the analysis. The final dataset contained of 248,475 sequences (Additional file 1).

**Parasite material and RNA extraction and RNA-seq data generation.** Adult *A. suum* and *T. suis* were obtained from swine infected as weanling pigs (mixed breed, Swine Center, Washington State University) 60 to 70 days, or 50 days, post-infection, respectively. Infections for *A. suum* were initiated with larvated eggs as described ([Jasmer et al., 2014](#_ENREF_9)). Infections of *T. suis* were initiated with 10,000 larvated eggs (provided by Dr. Joseph Urban, USDA Beltsville, MD) administered orally to each pig. Adult *H. contortus* were obtained at 28 days post-infection from lambs infected with 10,000 infective larvae, as described ([Jasmer et al., 1993](#_ENREF_10)). Parasite intestinal samples were dissected from two or more freshly isolated *A. suum*, or greater than 10 *T. suis* or *H. contortus* worms maintained in ice cold phosphate buffered saline (PBS, pH 7.4). Dissected intestines were placed immediately in Trizol for isolation of RNA, as we have done previously ([Jasmer et al., 2000](#_ENREF_11)). Samples from male and female worms were prepared separately for each species.

Non-normalized cDNA was used to construct Multiplexed Illumina paired-end small fragment libraries according to the manufacturer's recommendations (Illumina Inc, San Diego, CA), with the following exceptions: 1) 1 ug of cDNA was sheared using a Covaris S220 DNA Sonicator (Covaris, INC. Woburn, MA) to a size range between 200–400 bp. 2) Four rounds of PCR amplifications were performed to enrich for proper adapter ligated fragments and properly index the libraries. 3) The final size selection of the library was achieved by an AMPure paramagnetic bead cleanup (Agencourt, Beckman Coulter Genomics, Beverly, MA), targeting 300–500 bp. The concentration of the library was accurately determined through qPCR according to the manufacturer's protocol (Kapa Biosystems, Inc, Woburn, MA) to produce cluster counts appropriate for the Illumina platform. Multiple indexed libraries were pooled together and loaded into one lane of a HiSeq2000 version 3 flow cell. 2x101 bp read pairs (later clipped to 100 bp using Consensus Assessment of Sequence and Variation [CASAVA, version 1.8]) were generated for each sample. Male and female intestinal samples were sequenced using the same protocol.

**Analytical processing and mapping of the RNA-seq reads.** Analytical processing of the Illumina short-reads was performed using in-house scripts. DUST([Morgulis et al., 2006](#_ENREF_17)) was used to filter out regions of low compositional complexity and to convert them into N's. An in-house script was used to remove N's, and discard reads without at least 60 bases of non-N sequence. Gene expression for each sample was calculated by mapping the screened RNA-Seq reads to the coding DNA sequences (CDS) of the three species respectively using Tophat ([Trapnell et al., 2009](#_ENREF_21)) (version 2.0.8), and calculating depth and breadth of coverage per gene using Refcov (version 0.3, <http://gmt.genome.wustl.edu/gmt-refcov/>). Genes with 50% or more breadth of coverage were considered to be expressed in the intestine of each species. The union of those genes expressed in male or female sample was identified as the list of genes expressed in the intestine for each species.

**Detection of protein family death/birth and protein Sequence duplication/deletion.** Protein family death and birth events, as well as duplication and deletion events of protein sequences within protein families, were identified as previously described ([Wang et al., 2012](#_ENREF_23)). Briefly, protein family death and birth were evaluated using DOLLOP in the phylogeny inferring package PHYLIP ([Felsenstein, 2005](#_ENREF_5)). DOLLOP reconstructed the ancestral states for all the characters (protein families) using a dollo parsimony algorithm([Le Quesne, 1974](#_ENREF_15)). Protein family deaths and births were inferred by checking the states of these characters on each lineage of the 15 species tree. Duplication and deletion events of intestinal protein family members were quantified using Urec([Gorecki and Tiuryn, 2007](#_ENREF_7)). First, the sequences for each family were aligned using MUSCLE ([Edgar, 2004](#_ENREF_4)). The distance matrices and reconstructed phylogenetic trees for each set of aligned sequences were computed using PRODIST and NEIGHBOR of PHYLIP, respectively. The reconstructed trees were reconciled with the species trees of the 15 taxa to infer the duplication and deletion events over their evolution using Urec. The relative rates of the corresponding events of each lineage were computed by normalizing the numbers of events using inferred branch lengths. These inferred branch lengths were derived from the multiple alignments of all universal families with using PROMLK of PHYLIP (http://evolution.genetics.washington.edu/phylip/doc/promlk.html).

To illustrate the changes of organism complexity over the course of evolution, we computed indices defining protein family change and adaptation. In summary, the log ratio of birth and death events (log(B/D)) of protein families at different lineages was defined as the protein family change index (PFCI). For each lineage, if the birth events outnumber death events, the index is larger than 0, suggesting that the organism has gained complexity over the lineage, and vice versa ([Wang et al., 2012](#_ENREF_23)). The calculations were carried out for both all protein families derived from whole proteomes as well as the intestinal protein families derived from transcriptomes (where applicable) to quantitatively assess protein evolution associated with speciation under both conditions.

**Indel detection and structure localization.** Nematode-specific insertions and deletions in the protein sequences were identified as previously reported([Wang et al., 2009](#_ENREF_22)). Briefly, protein families were first split into nematode families (NemFam) and reference families (RefFam) (host and outgroups). The RefFam sequences were aligned first utilizing MUSCLE ([Edgar, 2004](#_ENREF_4)). The NemFam sequences were then aligned to improved RefFam alignments using the profile alignment function of CLUSTALW ([Thompson et al., 1994](#_ENREF_20)). After alignment, the NemFam and RefFam sequences were split into separate files and the NemFam sequences were manually curated to improve alignment and reduce redundancy. The resulting improved alignments were used for detection of insertions and deletions. A gap absent from the RefFam sequences was recorded as being a ‘nematode specific deletion’, while gaps present only in the RefFam sequences were recorded as a ‘nematode specific insertion’.

# 3. Supplementary References

Benson, D.A., Karsch-Mizrachi, I., Clark, K., Lipman, D.J., Ostell, J., and Sayers, E.W. (2012). GenBank. Nucleic Acids Res 40, D48-53.

Bernstein, F.C., Koetzle, T.F., Williams, G.J., Meyer, E.F., Jr., Brice, M.D., Rodgers, J.R., Kennard, O., Shimanouchi, T., and Tasumi, M. (1977). The Protein Data Bank: a computer-based archival file for macromolecular structures. Journal of molecular biology 112, 535-542.

Desjardins, C.A., Cerqueira, G.C., Goldberg, J.M., Dunning Hotopp, J.C., Haas, B.J., Zucker, J., Ribeiro, J.M., Saif, S., Levin, J.Z., Fan, L., et al. (2013). Genomics of Loa loa, a Wolbachia-free filarial parasite of humans. Nat Genet 45, 495-500.

Edgar, R.C. (2004). MUSCLE: multiple sequence alignment with high accuracy and high throughput. Nucleic acids research 32, 1792-1797.

Felsenstein, J. (2005). PHYLIP (Phylogeny Inference Package) (Seattle, WA, USA: Department of Genome Sciences, University of Washington), p. Distributed by the author.

Ghersi, D., and Sanchez, R. (2009). EasyMIFS and SiteHound: a toolkit for the identification of ligand-binding sites in protein structures. Bioinformatics 25, 3185-3186.

Gorecki, P., and Tiuryn, J. (2007). URec: a system for unrooted reconciliation. Bioinformatics 23, 511-512.

Harris, T.W., Baran, J., Bieri, T., Cabunoc, A., Chan, J., Chen, W.J., Davis, P., Done, J., Grove, C., Howe, K., et al. (2013). WormBase 2014: new views of curated biology. Nucleic Acids Res.

Jasmer, D., Rosa, B., and Mitreva, M. (2014). Peptidases compartmentalized to the Ascaris suum intestinal lumen and apical intestinal membrane. . PLoS Negl Trop Dis Accepted for publication.

Jasmer, D.P., Perryman, L.E., Conder, G.A., Crow, S., and McGuire, T. (1993). Protective immunity to Haemonchus contortus induced by immunoaffinity isolated antigens that share a phylogenetically conserved carbohydrate gut surface epitope. J Immunol 151, 5450-5460.

Jasmer, D.P., Yao, C., Rehman, A., and Johnson, S. (2000). Multiple lethal effects induced by a benzimidazole anthelmintic in the anterior intestine of the nematode Haemonchus contortus. Molecular and biochemical parasitology 105, 81-90.

Jex, A.R., Liu, S., Li, B., Young, N.D., Hall, R.S., Li, Y., Yang, L., Zeng, N., Xu, X., Xiong, Z., et al. (2011). Ascaris suum draft genome. Nature 479, 529-533.

Kinsella, R.J., Kahari, A., Haider, S., Zamora, J., Proctor, G., Spudich, G., Almeida-King, J., Staines, D., Derwent, P., Kerhornou, A., et al. (2011). Ensembl BioMarts: a hub for data retrieval across taxonomic space. Database : the journal of biological databases and curation 2011, bar030.

Laing, R., Kikuchi, T., Martinelli, A., Tsai, I.J., Beech, R.N., Redman, E., Holroyd, N., Bartley, D.J., Beasley, H., Britton, C., et al. (2013). The genome and transcriptome of Haemonchus contortus, a key model parasite for drug and vaccine discovery. Genome Biol 14, R88.

Le Quesne, W.J. (1974). The Uniquely Evolved Character Concept and its Cladistic Application. Systematic Zoology 23, 513-517.

Mitreva, M., Jasmer, D.P., Zarlenga, D.S., Wang, Z., Abubucker, S., Martin, J., Taylor, C.M., Yin, Y., Fulton, L., Minx, P., et al. (2011). The draft genome of the parasitic nematode Trichinella spiralis. Nat Genet 43, 228-235.

Morgulis, A., Gertz, E.M., Schaffer, A.A., and Agarwala, R. (2006). A fast and symmetric DUST implementation to mask low-complexity DNA sequences. Journal of computational biology : a journal of computational molecular cell biology 13, 1028-1040.

Roy, A., Kucukural, A., and Zhang, Y. (2010). I-TASSER: a unified platform for automated protein structure and function prediction. Nature protocols 5, 725-738.

Tang, Y.T., Gao, X., Rosa, B.A., Abubucker, S., Hallsworth-Pepin, K., Martin, J., Tyagi, R., Heizer, E., Zhang, X., Bhonagiri-Palsikar, V., et al. (2014). Genome of the human hookworm Necator americanus. Nature genetics.

Thompson, J.D., Higgins, D.G., and Gibson, T.J. (1994). CLUSTAL W: improving the sensitivity of progressive multiple sequence alignment through sequence weighting, position-specific gap penalties and weight matrix choice. Nucleic Acids Res 22, 4673-4680.

Trapnell, C., Pachter, L., and Salzberg, S.L. (2009). TopHat: discovering splice junctions with RNA-Seq. Bioinformatics 25, 1105-1111.

Wang, Z., Martin, J., Abubucker, S., Yin, Y., Gasser, R.B., and Mitreva, M. (2009). Systematic analysis of insertions and deletions specific to nematode proteins and their proposed functional and evolutionary relevance. BMC Evol Biol 9, 23.

Wang, Z., Zarlenga, D., Martin, J., Abubucker, S., and Mitreva, M. (2012). Exploring metazoan evolution through dynamic and holistic changes in protein families and domains. BMC Evol Biol 12, 138.
